# In-situ start and end of growing season dates of major European crop types from France and Bulgaria at a field level

**DOI:** 10.1016/j.dib.2023.109623

**Published:** 2023-09-27

**Authors:** Dessislava Ganeva, Tiphaine Tallec, Aurore Brut, Egor Prikaziuk, Enrico Tomelleri, Gerbrand Koren, Jochem Verrelst, Katja Berger, Lukas Valentin Graf, Santiago Belda, Zhanzhang Cai, Cláudio F. Silva

**Affiliations:** a Space Research and Technology Institute, Bulgarian Academy of Sciences, Acad. Georgi Bonchev bl.1, 1113 Sofia, Bulgaria; b CESBIO, Université de Toulouse, CNES/CNRS/INRAE/IRD/UPS, Toulouse, France; c Faculty of Geo-Information Science and Earth Observation (ITC), University of Twente, 7500 AE Enschede, The Netherlands; d Faculty of Agricultural Environmental and Food Sciences Free University of Bozen/Bolzano, 39100 Bolzano, Italy; e Copernicus Institute of Sustainable Development, Utrecht University, Princetonlaan 8a, 3584 CB Utrecht, The Netherlands; f Image Processing Laboratory (IPL), University of Valencia, 46980 Paterna, Spain; g Mantle Labs GmbH, Vienna, Austria; h Institute for Agricultural Science, ETH Zürich, Universitatsstrasse 2, CH-8092 Zürich, Switzerland; i Earth Observation of Agroecosystems Team, Division Agroecology and Environment, Agroscope, Reckenholzstrasse 191, CH-8042 Zurich, Switzerland; j Department of Applied Mathematics, Universidad de Alicante, Carretera San Vicente del Raspeig s/n 03690 San Vicente del Raspeig - Alicante, Spain; k Department of Physical Geography and Ecosystem Science, Lund University, Solvegatan 12, S-223 62 Lund, Sweden; l Forest Research Centre (CEF) and Associated Laboratory TERRA, School of Agriculture, University of Lisbon, Tapada da Ajuda, 1349-017 Lisbon, Portugal

**Keywords:** Winter crop, Summer crop, Cover crop, BBCH, Phenology

## Abstract

Crop phenology data offer crucial information for crop yield estimation, agricultural management, and assessment of agroecosystems. Such information becomes more important in the context of increasing year-to-year climatic variability. The dataset provides in-situ crop phenology data (first leaves emergence and harvest date) of major European crops (wheat, corn, sunflower, rapeseed) from seventeen field study sites in Bulgaria and two in France. Additional information such as the sowing date, area of each site, coordinates, method and equipment used for phenophase data estimation, and photos of the France sites are also provided. The georeferenced ground-truth dataset provides a solid base for a better understanding of crop growth and can be used to validate the retrieval of phenological stages from remote sensing data.

Specifications Table

**Table:** 

Subject	Agricultural Sciences: Agronomy and Crop Science		
Specific subject area	In-situ start and end of growing season data of numerous crop types at the field level		
Type of data	Two Tables (text format), one with the crop phenology data and the additional information of each site and another with the description of each column variable of the crop phenology data. Georeferenced plots from all sites (GeoJSON file format). Open-format georeferenced plot data from all sites (CSV file format). One file (text format), with support data for the georeferenced files. Photos from French sites (JPEG file format).		
How the data were acquired	The sowing and harvest date were previously communicated to the researchers. The phenology in-situ data were acquired with systematic field observations by an agronomist, pictures taken by a NetCam SC IR (StarDot Technologies, Buena Park, CA) camera (phenocam) and digital photo camera (RICOH G700SE, RICOH International, Düsseldorf, Germany). The first leave emergence was determined in relation to the homogeneity or heterogeneity of the cover to which an average BBCH stage was attributed using a BBCH-scale which is traditionally used to identify the phenological development stages of plants.		
Data format	Raw data		
Description of data collection	We monitored seventeen production fields in Bulgaria (one growing season each) and two research fields in France (8 – 10 growing seasons from 2015 – 2022). The in-situ data were collected at a field level and consisted of sowing date, first leaves emergence (phenophase, BBCH10-BBCH13 date), and harvest date.		
Data source location	Private agriculture companies and cooperatives Dobrich Bulgaria		

	Field	Latitude (°N)	Longitude (°E)

	P0	43.4676	27.6658
	P01	43.5027	28.2244
	P02	43.5624	28.2005
	P1	43.4837	27.6311
	P2	43.4638	27.6174
	P3	43.4726	27.6831
	P4	43.5263	27.8149
	P13	43.4931	28.2323
	P14	43.4317	27.2595
	P15	43.2926	27.4952
	P17	43.4441	27.6366
	P18	43.5295	27.8140
	P19	43.4721	28.2294
	P20	43.5099	28.2374
	P22	43.5018	28.2771
	P23	43.4752	28.2714
	P24	43.4842	28.2784
	CESBIO (UT3, CNES, CNRS, INRAE, IRD) Auradé and Lamasquère France		

	Field	Latitude (°N)	Longitude (°E)

	FR-Aur	43.5495	1.1061
	FR-Lam	43.4992	1.2358
Data accessibility	Repository name: Zenodo Data identification number: 8067432 Direct URL to data: https://doi.org/10.5281/zenodo.8067432		

## 1 Value of the Data

• In-situ start and end of growing season measurements at the field level are valuable because they offer direct ground-based observations on the timing and progression of crop growth stages [1].
• Improve the accuracy in identifying crop phenology stages [2].
• Develop more accurate models and forecasts, assess agroecosystems, and understand how seasonal cycles may be altered by climate change [3].
• These data are highly valuable to ecologists, agronomists, and modelers, as the recent EU dataset [4] is deficient in providing such information that could be beneficial for their work.
• When integrated with environmental and climatic data, crop phenology data can contribute to the study of climate impacts on agriculture, ultimately helping to optimize or adjust crop production and management [5].
• It can also serve as a reliable ground validation for remote sensing studies and provide valuable data to optimize phenology extraction models.

## 2 Objective

Our dataset [6] offers precise, field-level information on the timing of two critical growth stages: the start and end of the growing season. It is designed to advance research in land surface phenology (LSP) by enabling better understanding and prediction of crop phenology patterns. This knowledge is crucial for various purposes, including optimizing planting and harvesting schedules, managing crop health, and improving overall agricultural productivity.

The dataset serves as a resource for researchers, policymakers, and stakeholders in the agricultural sector. By analyzing the timing and duration of the growing season, we can enhance our understanding of crop phenology variations. This understanding allows for better predictions and informed decision-making regarding agricultural practices.

By maximizing the utility of existing data, we can contribute to the advancement of crop phenology research and support sustainable and resilient agricultural practices.

## 3 Data Description

**Fig. 1:**
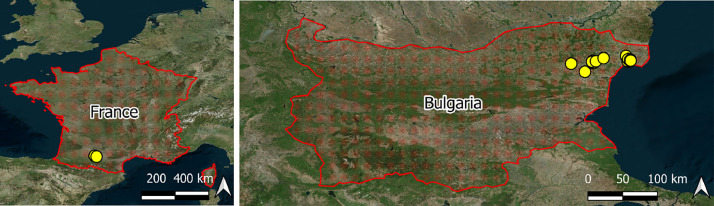
The location of study fields (yellow dots) where the in-situ crop phenology stages were determined and acquired. Background - google satellite image.

We provide an in-situ crop phenology stages dataset from two different countries, Bulgaria and France, at a field level, see Fig. 1.

**Fig. 2:**
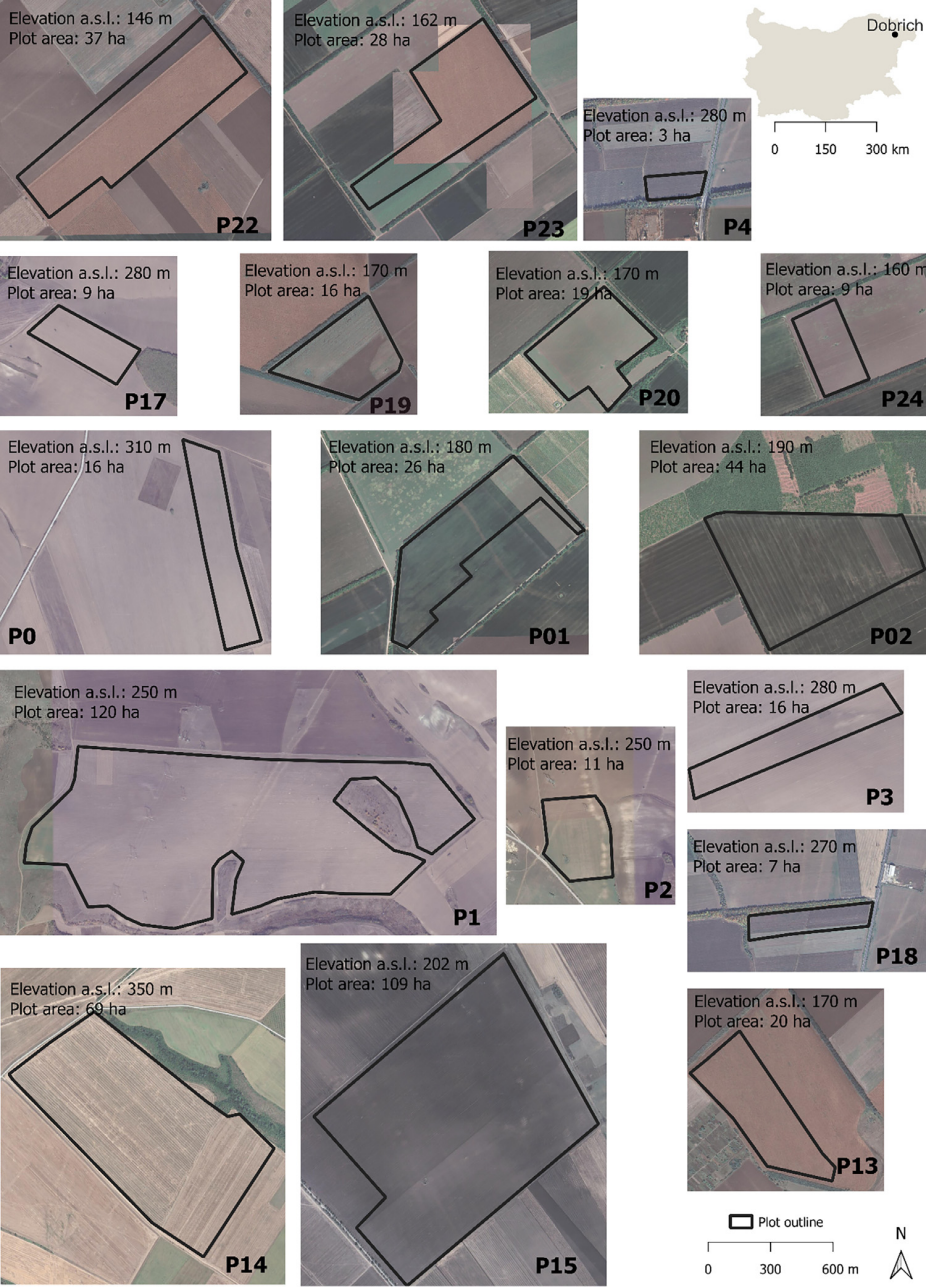
Shape of the selected seventeen field study sites in Bulgaria, P0, P01, P02, P1, P2, P3, P4, P13, P14, P15, P17, P18, P19, P20, P22, P23, and P24 with the corresponding elevation above sea level (a.s.l) in meters (m) and the area in hectares (ha). Each site is outlined. On the right are the map of Bulgaria and the name of the region where the sites are located. The scale bar in the top right refers to the image of the country and, in the bottom right it refers to the images of the field study sites.

**Fig. 3:**
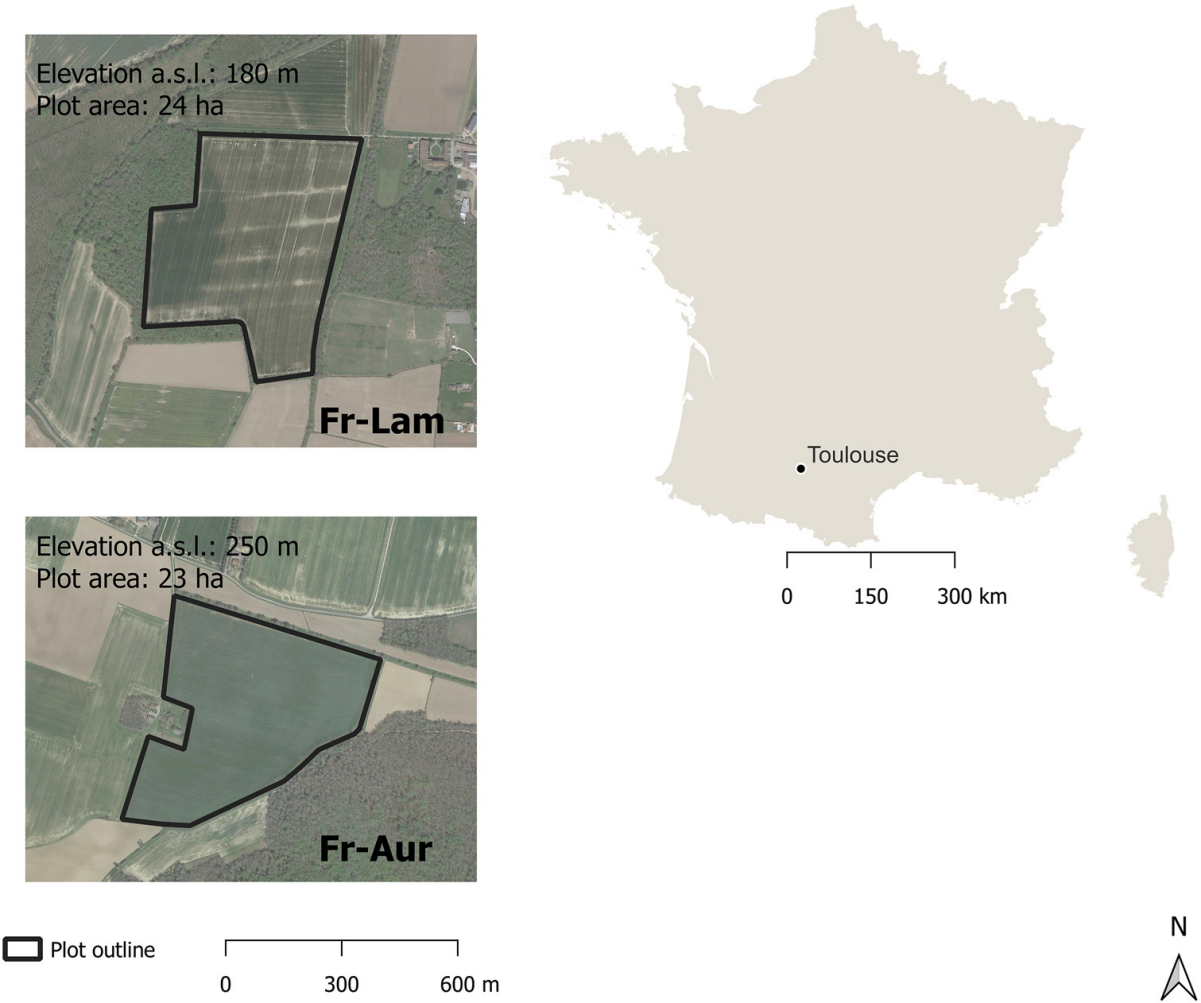
Shape of the selected field study sites in France, Auradé (FR-Aur) and Lamasquère (FR-Lam) on the left with the corresponding elevation above sea level (a.s.l) in meters (m) and the area in hectares (ha). Each site is outlined. On the top right are the map of France and the name of the city where the sites are located. The scale bar on the right of the figure refers to the image of the country and, in the left bottom it refers to the images of the field study sites.

In Bulgaria, we monitored seventeen fields, Fig. 2, and in France two fields, Fig. 3.

The collected data set runs from the growing season 2015/2016 until 2018/2019 for Bulgarian field sites and 2015/2016 until 2021/2022 for France field sites, comprising a total of 35 observations (rows). This dataset contains information on phenology stages (sowing date, first leaves emergence/phenophase data, BBCH10-BBCH13, date, and harvest date), method and equipment used to access the phenophase, area, coordinates, crop type and the name of the associated photos of the France sites and the georeference data of each field site (file name: 1_senseco_data_insitu_crop_phenology_Bulgaria_France.txt). See the details of the file in Table 1. The photos of the France field sites are in JPG format. All field study sites from both countries are georeferenced in GeoJSON and CSV format. There is a metadata file (metadata_geojson_csv.txt) with support information for the field study sites of both countries related to the GeoJSON and CSV format files.

**Table 1:** Field identification and in-situ data structure: variables, type, descriptions, and unit or category at field level. Missing values represented by ‘NA’ (txt format).

Variable	Type	Description	Unit or category
record	numeric	data numeric record	sequential numbers
country	character	country of the crop observations	i.e. France
site	character	name of the site of the crop observations	i.e. Gurkovo
plot_ID	character	unique code associated with each crop plot	i.e. P3
area	numeric	area of each crop plot	ha (hectares)
latitude	numeric	latitude coordinate of the crop plot center	decimal format
longitude	numeric	longitude coordinate of the crop plot center	decimal format
crop_type	character	common name of each crop	i.e. winter Rapeseed
crop_genus	character	genus name of each crop	i.e. Triticum
season	date	season of each crop	year/year
sowing_date	date	sowing date of each crop plot and season	year/month/day
harvest_date	date	harvest date of each crop plot and season	year/month/day
phenophase_date	date	date of the selected phenophase of each crop plot and season	year/month/day
phenophase	character	stage of each crop in the corresponding “phenophase_date”	First leaves emergence (BBCH10-13)
method_phenophase	character	method used to access the phenophase of each crop plot and season	i.e. observation on site
equipment_method_phenophase	character	equipment used to access the phenophase of each crop plot and season	i.e. phenocam
photo_available	character	if photo(s) from each crop plot and season is/are available	yes or no
photo_ID	character	unique code for each photo for each crop plot and season	i.e. FR-Aur_Sunflower_20200507
plot_shape_ID	character	unique code for each georeferenced crop plot and season	i.e. Plot_P18

On the Zenodo repository, there is one metadata file (2_senseco_metadata_insitu_crop_phenology_Bulgaria_France.txt) with columns description of the actual dataset (1_senseco_data_insitu_crop_phenology_Bulgaria_France.txt).

The main phenology information consists of sowing date, phenophase (BBCH10-13) data, date, and harvest date at a field level. Phenophase date corresponds to the first leave emergence characterized by BBCH10-13 distinguishable phenology stage.

## 4 Experimental Design, Materials and Methods

### 4.1 Site description

The Bulgarian fields Oborishte (P0 and P3), Gurkovo (P01 and P13), Trigorci (P02), General Kiselovo (P1 and P2), Dobrich (P4 and P18), Mirovci (P14), Neofit Rilski (P15), Boyana (P17) and Gurkovo (P19, P20, P22, P23, P24) are situated in the Danube plain, Northeast part of Bulgaria, in Dobrich area (Fig. 2). The area is mostly flat, the soil has mainly sandy loam texture, the climate in this region is Moderate Continental with cold winters and hot summers (mean daily temperature 10.2°C), and an annual cumulative rainfall of 540 mm.

The French sites are located in Southwestern France near Toulouse (Fig. 3). The straight line distance between both sites is 12 kilometers. Both experimental plots are part of the Regional Spatial Observatory South West (OSR SW), the regional Zone Atelier Pyrénées-Garonne (ZA PYGAR, [7]), the national research infrastructure Critical Zone Observatories: Research and Applications (OZCAR; [8]) and the Integrated Carbon Observation System (ICOS; [9]) European network.

The FR-Lam field is part of an experimental dairy farm (Domaine de Lamothe, Ecole d'ingénieurs de Purpan) located in a plain where a winter wheat – irrigated maize crop rotation is performed. The crops are grown for feed and are fertilized with dairy excreta or slurry. The silage maize is harvested when the plant is still green, and the kernels are not yet ripe (i.e., between the middle and end of August). The FR-Lam site is located in a vulnerable zone, which means that it is subject to nitrate leaching. Within the framework of the 4th nitrate directive, issued from a European regulatory framework, farmers are obliged to cover the soil of agricultural plots located in these zones during the winter with the introduction of cover crops (i.e. white mustard, vetch, faba bean, and phacelia) between a winter crop and a summer crop.

The FR-Aur crop site is part of a grain farm located in a hilly area where a winter wheat – rapeseed –barley–sunflower crop rotation is performed. The soils of agricultural plots in this rugged landscape are often subject to erosion during periods of bare soil and intense rainfall. Therefore, to limit the loss of soil from their plots, farmers introduce intermediate cover crops during the winter between a winter and a summer crop.

On both French sites cover crops are crushed green and incorporated into the soil, not exported.

The climate on both sites is a temperate climate with oceanic and Mediterranean influences, with mild winters, rainy springs, and very hot summers with very low rainfall, followed by very sunny autumns. From a nearby MeteoFrance measurement station (Lherm-Muret), the mean annual rainfall calculated over the past 24 years was 617 ± 101 mm and the mean annual temperature was 13.7 ± 0.6°C on both sites FR-Lam and FR-Aur [10,11].

According to the textural triangle of [12], the soil of FR-Lam and FR-Aur sites are clay-silty (50.3% clay, 35.8% silt, 11.2% sand, 2.8% organic matter) and clayey to sandy-clay (30.8% clay, 48.3% silt, 19.2% sands, 1.6% organic matter) respectively.

### 4.2 Data acquisition

The parcel shape was acquired by manual outlining of each field in a Geographical Information System (GIS) software. The parcel area was computed by geometry calculator. The latitude and longitude of each field were acquired by generating a field centroid and extracting its coordinates.

Usually, with parcel-level phenological estimates, it is important to acknowledge that the parcel is rarely in a uniform phenological stage, as different parts of the parcel may exhibit varying growth rates. This variability requires the use of the 50% threshold rule to determine the overall phenological stage of the field. This widely employed approach in agricultural and ecological research provides a practical and standardized method for assessing the phenological stage of a field [13].

In this study, the dates of emergence for all fields were determined using the 50% threshold rule. This rule is a simplification that allows for considering the entire parcel as being in a uniform phenological stage. While acknowledging the inherent variability within the parcel, applying the 50% threshold rule by both an agronomist in the field and through digital camera picture (phenocam and digital photo camera) analysis ensures a common collection protocol.

The agronomist's observation footprint extends from the immediate proximity of the edge of the plot to a distant view of the entire plot, encompassing both close-up and panoramic perspectives. The phenocam observation footprint covers approximately 5 to 10% of the plot. The image analysis entailed examining both the immediate close range and the distant areas of the plot.

The agronomist assessed the phenophase (emergence) during the moment of observation, while the analysis of photos involved evaluating the phenophase at the time of analysis. Despite these differences, the agronomist and images observations share sufficient similarities to enable comparability. The phenological development stage was identified using the BBCH-scale.

The harvest date was recorded as the start date of the harvest.

In Bulgaria, crop data (sowing date, phenophase data, and harvest date) from seventeen fields were collected by visual observations, written in a paper notebook, and subsequently passed to an Excel file. The available data for each field is from one growing season and thirteen fields miss phenophase data or harvest date.

Bulgarian fields are production fields cultivated by companies or cooperatives. Each field has a dedicated agronomist that provides the sowing date, emergence phenophase (date, and harvest date. The agronomist frequently visits the field and evaluted the phenological stage of the field. In certain years, winter crops faced unfavorable growing conditions, such as drought during crop emergence. A decision is then made after the winter period whether to keep or destroy the crop. Consequently, for some fields that have been planted with winter crops, only a portion of the crop may survive, with the rest being destroyed. The dataset provides information on the shape and area of the remaining crop that has been kept until harvest. The collection of the dataset took place during Dessislava Ganeva PhD research, which had a different objective than phenology monitoring. As a result, some fields within the dataset lack emergence dates or harvest dates. Nevertheless, we made the decision to include these plots with incomplete data in the proposed dataset due to the valuable information they still provide.

In France, crop data (sowing date and harvest date) from 2 fields (FR-Aur and FR-Lam) were collected through visual observation, identical to the Bulgaria sites. The phenophase data was collected by field observations, phenocam picture and/or digital photo camera picture analysis. The exception is for the FR-Lam site during the season 2017/2018 and 2019/2020 where the phenophase data was collected only through field observations, identical to the Bulgaria sites. The available data for both fields cover numerous growing seasons.

## Ethics Statements

The current work meets the ethical requirements for publication in Data in Brief and does not involve human subjects, animal experiments, or any data collected from social media platforms.

Proper permissions were taken from the private farm companies for collecting data from their land and their data distribution policies complied with.

## CRediT authorship contribution statement

**Dessislava Ganeva:** Conceptualization, Methodology, Validation, Investigation, Resources, Writing – review & editing, Supervision, Project administration. **Tiphaine Tallec:** Methodology, Validation, Investigation, Resources, Writing – review & editing, Project administration. **Aurore Brut:** Methodology, Validation. **Egor Prikaziuk:** Software, Data curation, Writing – review & editing. **Enrico Tomelleri:** Writing – review & editing. **Gerbrand Koren:** Writing – review & editing, Software, Data curation. **Jochem Verrelst:** Funding acquisition. **Katja Berger:** Funding acquisition. **Lukas Valentin Graf:** Writing – review & editing. **Santiago Belda:** Writing – review & editing. **Zhanzhang Cai:** Writing – review & editing. **Cláudio F. Silva:** Conceptualization, Data curation, Writing – original draft, Visualization.

## Data Availability

In-situ crop phenology dataset from sites in Bulgaria and France (Original data) (Zenodo)
